# Computer-aided assessment of regional vascularity of thyroid nodules for prediction of malignancy

**DOI:** 10.1038/s41598-017-14432-7

**Published:** 2017-10-30

**Authors:** Faisal N. Baig, Jurgen T. J. van Lunenburg, Shirley Y. W. Liu, Shea-Ping Yip, Helen K. W. Law, Michael Ying

**Affiliations:** 1 Department of Health Technology and Informatics, The Hong Kong Polytechnic University, Hung Hom, Kowloon, Hong Kong, SAR China; 2 Department of Surgery, Prince of Wales Hospital, The Chinese University of Hong Kong, Shatin, New Territories, Hong Kong, SAR China

## Abstract

Color Doppler vascular index (VI) was assessed alone and in combination with grey-scale ultrasound (GSU) in regionally subdivided thyroid nodules in diagnosing thyroid cancer. Color Doppler sonograms of 111 thyroid nodules were evaluated by a home-developed algorithm that performed “offsetting” (algorithm for changing the area of a region of interest, ROI, without distorting the ROI’s contour) and assessed peripheral, central and overall VI of thyroid nodules. Results showed that the optimum offset for dividing peripheral and central regions of nodule was 22%. At the optimum offset, the mean VI of peripheral, central, and overall regions of malignant nodules were significantly higher than those of benign nodules (26.5 ± 16.2%, 21.7 ± 19.6%, 23.8 ± 4.6% v/s 18.2 ± 16.7%, 11.9 ± 15.1% and 16.6 ± 1.8% respectively, *P* < 0.05). The optimum cut-off of peripheral, central, and overall VI was 19.7%, 9.1% and 20.2% respectively. When compared to GSU alone, combination of VI assessment with GSU evaluation of thyroid nodules increased the diagnostic accuracy from 58.6% to 79.3% (*P* < 0.05). In conclusion, a novel algorithm for regional subdivision and quantification of thyroid nodular VI in ultrasound images was established, and the optimum offset and cut-off were derived. Assessment of intranodular VI in conjunction with GSU can increase the accuracy in ultrasound diagnosis of thyroid cancer.

## Introduction

Thyroid cancer accounts for about 3.8% of all malignant cases^1^. Among endocrine malignancies, thyroid cancer ranks the highest in the incidence. According to the National Institute of Cancer in the USA, there are 64,330 new cases of thyroid cancer in 2016^1^. Fine needle aspiration cytology (FNAC) is the current standard method to diagnose thyroid cancer but it has varied range of specificity (74–92%) and the sensitivity is low (66%)^2^. In addition, about 20–30% of FNAC results are non-diagnostic^3^. Grey-scale ultrasound (GSU) is commonly used to assess thyroid nodules and various GSU features for identifying malignant nodules have been reported such as microcalcification, hypoechogenicity, absent halo sign, solid internal structure, and irregular margins^4–7^. However, assessment of these features is subjective and qualitative which is vulnerable to inter and intra-observer variability. In addition, none of the GSU feature is specific to malignancy^8^.

Angiogenesis is an important means of proliferation in neoplastic cells and is associated with malignancy^9,10^. Doppler ultrasound has potential to detect differences in blood flow of healthy organs and cancerous tissues. Color Doppler and power Doppler ultrasound have been used in the differential diagnosis of breast cancer, ovarian cancer, hepatic cancer, and prostate cancer^8,11–14^. Color Doppler ultrasound assessment of thyroid cancer has been reported. Results of some studies suggested that color Doppler ultrasound is useful in differentiation between benign and malignant thyroid nodules^15–17^, whilst others suggested that Doppler ultrasound has limited value in prediction of thyroid malignancy^18–23^. These controversial findings are probably due to the fact that assessment of thyroid nodular vascularity was performed subjectively rather than using an objective and quantitative approach in previous studies. This leads to high inter and intra-observer variability in the assessment of thyroid nodular vascularity^20^. Previous reports suggested that hypervascularity of thyroid nodule is a useful predictor of thyroid malignancy. However, in previous studies diagnosis of thyroid cancer was based on visual detection of increased vascularity in thyroid nodule which has potential risk of inter and intra observer variability^2,4,23,24^.

In our previous studies, we have developed a computer-aided approach and applied it in the assessment and quantification of thyroid parenchymal vascularity and lymph node vascularity in color or power Doppler ultrasound images^21,25,26^. In color or power Doppler ultrasound assessment of thyroid nodule vascularity, peripheral vascularity is usually considered as a predictor of benignity and central vascularity tends to be associated with thyroid malignancy^16,17^. However, previous studies used subjective and qualitative methods to assess the vascular distribution in peripheral and central regions of thyroid nodules which is not accurate and reliable. Therefore, the aim of the present study was to modify our previously reported computer-aided method so that it can automatically evaluate and quantify the vascularity of the peripheral and central regions of thyroid nodules. The study also aimed to evaluate the diagnostic accuracy of this computer-aided detection (CAD) of regional vascularity in distinguishing benign and malignant thyroid nodules, and to assess the added value of this CAD approach on GSU assessment of thyroid nodules.

## Results

### Histopathology

The mean age of patients with benign nodules (n = 84) and of patients with malignant nodules (n = 27) were 51.2 ± 12 and 56.6 ± 17.6 years respectively (*P* > 0.05). In each patient, the thyroid nodule with confirmed cytological/histological result was included in the study. Among the 111 thyroid nodules in the 111 patients, 62 benign nodules were identified by fine needle aspiration cytology and the remaining 49 nodules had pathological evaluation after surgical resection. Among these 49 nodules, pathology results revealed 22 benign and 27 malignant nodules (23 papillary thyroid carcinomas, 3 follicular carcinomas and 1 Hurthle cell carcinoma).

### Grey scale ultrasound

GSU evaluation of 111 thyroid nodules showed that malignant nodules tended to have microcalcification (77.8%), hypoechoic (92.6%), irregular margins (55.6%) and tall to width ratio >1 (59.3%) whereas these features were less common in benign nodules (microcalcification, 7.1%; hypoechoic, 33.3%; irregular margins, 16.7%; tall to width ratio >1, 13.1%). The differences were statistically significant *(P* < *0.05)*.

### Color Doppler ultrasound

Result showed that the mean overall vascular index (VI) of malignant nodules (23.8 ± 4.6%) was significantly higher than that of benign nodules (16.6 ± 1.8%) (*P* < 0.05). Table 1 shows the mean VI of the peripheral and central regions of benign and malignant nodules at different offset levels. When the offset level increased from 5% to 20%, the mean VI of the central region of malignant nodules was significantly higher than that of the benign nodules (*P* < 0.05), whereas there was no significant difference in the mean VI of the peripheral region of benign and malignant nodules (*P* > 0.05). On the contrary, at the offset level of 25%, the mean VI of the peripheral region of malignant nodules was significantly higher than that of the benign nodules (*P* < 0.05), whereas there was no significant difference in the mean VI of the central region of benign and malignant nodules (*P* > 0.05). In order to determine the optimum offset level, we evaluated the VI of the nodules at the offset levels of 21%, 22%, 23% and 24%. Results showed that at the offset level of 22% the mean VI of both central and peripheral regions of malignant nodules were significantly higher than those of benign nodules (*P* < 0.05), and thus the optimum offset level was 22%.

**Table 1:** Vascular index (VI) of peripheral and central regions of benign and malignant thyroid nodules at different offset levels.

Offset levels	Mean Peripheral VI ± SD(*P* value)	Mean Central VI ± SD(*P* value)
**5%-Offset**		
Benign	21.7 ± 20.3%	15.2 ± 15.3%
Malignant	23.5 ± 19.3%	23.4 ± 17.7%
	(*P* > *0.05*)	*(P* < 0.05)
**10%-Offset**		
Benign	21.4 ± 19.6%	13.5 ± 14.6%
Malignant	25.3 ± 18.7%	23.3 ± 18.0%
	(*P* > *0.05*)	*(P* < 0.05)
**15%-Offset**		
Benign	20.0 ± 18.4%	12.4 ± 14.5%
Malignant	24.0 ± 16.6%	22.3 ± 19.3%
	*(P* > *0.05*	*(P* < 0.05)
**20%-Offset**		
Benign	18.7 ± 17.1%	12.0 ± 14.7%
Malignant	25.0 ± 17.0%	21.6 ± 20.1%
	(*P* > *0.05*)	(*P* < 0.05)
**21%-Offset**		
Benign	18.4 ± 16.9%	11.9 ± 14.9%
Malignant	25.0 ± 17.1%	21.3 ± 20.1%
	*(P* > *0.05)*	*(P* < 0.05)
**22%-Offset**		
Benign	18.2 ± 16.7%	11.9 ± 15.1%
Malignant	26.5 ± 16.2%	21.7 ± 19.6%
	*(P* < 0.05)	*(P* < 0.05)
**23%-Offset**		
Benign	17.9 ± 16.5%	24.9 ± 17.1%
Malignant	11.9 ± 15.4%	20.9 ± 20.3%
	*(P* < 0.05)	*(P* > *0.05)*
**24%-Offset**		
Benign	17.8 ± 16.4%	11.9 ± 15.6%
Malignant	24.9 ± 17.2%	20.6 ± 20.1%
	*(P* < 0.05)	*(P* > *0.05)*
**25%-Offset**		
Benign	17.7 ± 16.3%	11.9 ± 16.0%
Malignant	26.6 ± 18.7%	20.4 ± 20.1%
	*(P* < 0.05)	*(P* > *0.05)*

Receiver operating characteristic (ROC) curves demonstrated the optimum cut-off values of VI when the overall VI, peripheral VI and central VI of the nodule were used to differentiate benign and malignant nodules, and the results are summarised in Table 2.

**Table 2:** Comparison of the diagnostic performance of peripheral VI, central VI and overall VI at 22% offset in distinguishing benign and malignant nodules.

Color Doppler VI at 22% Offset	Optimum Cut-off (%)	SEN (%)	SPEC (%)	NPV (%)	PPV (%)	Accuracy (%)
Peripheral VI	19.7	74.1	60.7	87.9	37.7	64.0
Central VI	9.1	74.1	60.7	87.9	37.7	64.0
Overall VI	20.2	74.1	69.0	89.2	43.5	70.3

VI, vascular index

SEN, sensitivity

SPEC, specificity

NPV, negative predictive value

PPV, positive predictive value.

Using the overall VI to distinguish benign and malignant nodules, it achieved diagnostic accuracy of 70.3% with sensitivity of 74.1%, specificity of 69%, negative predictive value (NPV) of 89.2% and positive predictive value (PPV) of 43.5% at the optimum cut-off level of 20.2 (Table 2).

At the 22% offset, both the peripheral VI and central VI had diagnostic accuracy of 64% with sensitivity of 74.1%, specificity of 60.7%, NPV of 87.9%, PPV of 37.7% at the optimum cut-off level of 19.7% and 9.1% respectively (Table 2).

**Figure 1:**
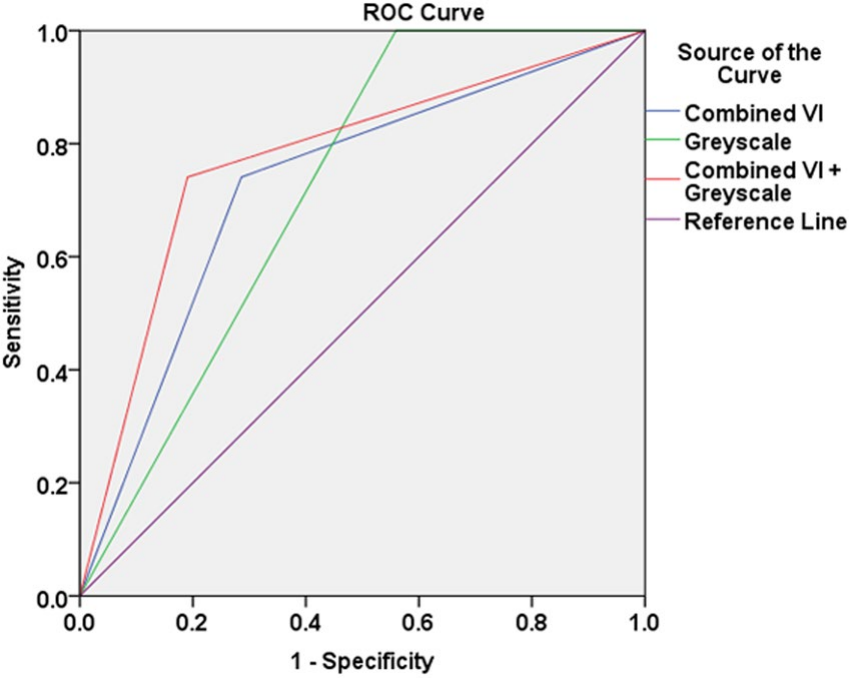
Receiver operating characteristic (ROC) curves show the diagnostic performance of grey scale ultrasound (Greyscale), combined peripheral, central and overall vascular indices assessment (Combined VI) and a combination of these two techniques (Combined VI + Greyscale) in the differentiation of benign and malignant thyroid nodules.

After determining optimum cut-off level of the peripheral, central, and overall VI, the criterion was set that a thyroid nodule was considered malignant when all three vascular indices (peripheral VI, central VI, and overall VI) are equal or greater than their respective cut-off values. True positive, true negative, false positive and false negative cases were evaluated. The diagnostic accuracy of the combined VI assessment in distinguishing benign and malignant nodules was 71.2% (Table 3, Fig. 1).

**Table 3:** Comparison between diagnostic performance of grey scale ultrasound, combined color Doppler vascular indices and their combination in the differentiation of benign and malignant thyroid nodules.

	SEN (%)	SPEC (%)	NPV (%)	PPV(%)	Accuracy (%)
GSU alone	96.3	46.4	97.5	36.6	58.6
Combined vascular indices	70.4	71.4	88.2	44.2	71.2
Combined vascular indices + GSU	66.7	83.3	88.6	56.3	79.3

GSU, grey scale ultrasound

SEN, sensitivity

SPEC, specificity

NPV, negative predictive value

PPV, positive predictive value.

In the evaluation of the diagnostic performance of GSU, a thyroid nodule was considered malignant when it presented with at least one of the suspicious GSU feature (i.e. microcalcification present, tall to width ratio >1, hypoechoic and irregular margins). The diagnostic accuracy of GSU in differentiating benign and malignant thyroid nodules was 58.6% (Table 3). The diagnostic performance of combining GSU and VI was determined based on the criterion that a thyroid nodule was considered to be malignant when it presented with at least one suspicious GSU feature and the combined VI was equal to or greater than the optimum cut-offs (i.e. peripheral VI > 19.7% and central VI > 9.1% and overall VI > 20.2%). Combination of color Doppler VI and GSU significantly increased the overall diagnostic accuracy from 58.6% to 79.3% *(P* < *0.05)* (Table 3, Fig. 1).

## Discussion

Angiogenesis has been reported as an important manifestation that promotes proliferation in cellular neoplasms and is linked to malignancy^27–29^. Increased vascularization in central region of thyroid nodules detected on Doppler ultrasound is an indication for fine needle aspiration cytology (FNAC) of the nodules, as recommended in guidelines provided by American Association of Clinical Endocrinologists (AACE), American College of Endocrinology (ACE) and Associazione Medici Endocrinologi (AME)^16,17,30,31^.

Frates *et al*.^16^ evaluated 254 thyroid nodules and found that the prevalence of malignancy was higher (41.9%) in hypervascular nodules, whereas only 14% of hypovascular malignant nodules were reported. Chan *et al*.^4^ evaluated vascularity in 55 cases of papillary thyroid carcinoma and found that 19% had central vascularity. Rago *et al*.^18^ evaluated the vascular pattern of benign and malignant thyroid nodules, and found that there was no significant difference between them. Moon *et al*.^20^ reported that assessment of thyroid nodular vascularity alone or in combination with GSU features was not as useful as GSU features alone in predicting thyroid malignancy. However, Brunese *et al*.^32^, Fobbe *et al*.^33^, Chammas *et al*.^34^ and Varverakis *et al*.^29^ found that intranodular vascularity was significantly associated with thyroid malignancy. The controversial findings of previous studies may be due to the reason that thyroid nodular vascularity was subjectively evaluated, and blood flow in peripheral and central regions of nodules was assessed on visual judgement rather than a standardised quantitative method. The visual assessment of intranodular vascularity was subjective and prone to inter and intra-observer variations and therefore the results may not be accurate.

In the present study, we have developed an innovative method that can automatically divide thyroid nodule into central and peripheral regions in sonogram, and compute the overall VI of the nodule as well as the regional VI of the peripheral and central areas of the nodule. Standardised and automated regional subdivision of thyroid nodules and quantification of regional vascularity of nodules are useful to eliminate human errors and variances in the assessment of intranodular vascularity of thyroid nodules. In this study, we found that 22% offset level is optimum in the differentiation of benign and malignant nodules. To the best of our knowledge, this is the first study that uses computer-aided method to subdivide thyroid nodule into different regions in ultrasound images, and quantify the regional vascularity of the nodule.

Lyshchik *et al*.^28^ analysed power Doppler images of 86 thyroid nodules and found significant increase in central vascularity of benign nodules with increase in tumor size. The authors found that the sensitivity and specificity in assessing intranodular vascularity varied with the size of the thyroid nodule, and they suggested that quantitative analysis is useful for assessing nodules smaller than 2 cm. However, only the overall vascularity of the nodules was evaluated in the study whereas regional subdivision of nodule and analysis of regional vascularity were not performed in the study^28^.

Sultan *et al*.^35^ analysed 100 thyroid nodules and quantified the vascular fraction area, mean flow velocity index and flow volume index of thyroid nodules in Doppler ultrasound images. Without assessing the diagnostic accuracy to determine the optimum separation of different regions of nodule, they manually segmented thyroid nodule into three equal sections (peripheral, intranodular and surrounding parenchyma). The authors concluded that quantitative evaluation of central vascularity of thyroid nodules has higher value in differential diagnosis of benign and malignant nodules. In Sultan *et al*.^35^, manual segmentation was used to divide the nodule into different regions. The method was subjective and prone to have intra and inter-operator variations. In comparison to Sultan *et al*.^35^ we adopted more standardized method by using a dedicated algorithm that uses the optimum offset level to accurately and objectively divide the thyroid nodule into peripheral and central regions. Using the same optimum offset level, different thyroid nodules can be assessed consistently and same regional division of nodule can be performed accurately and objectively. The new approach developed in the present study is more robust, objective, and highly reproducible with quantitative evaluation of vascularity that might have missed with visual inspection method.

In this study, there were 27 malignant and 84 benign thyroid nodules. The calculated power of this sample size in assessing the diagnostic performance of regional vascularity of thyroid nodule in distinguishing malignant and benign nodules ranged between 0.62 and 0.71. Further study with a larger sample size is suggested. Besides of the sample size, there are other limitations in the present study. Among the malignant nodules in the study, majority of them were papillary thyroid carcinoma (n = 23) and only 3 cases were follicular carcinoma and one Hurtle cell carcinoma. The value of assessing intranodular VI in distinguishing different types of malignant nodule was not conducted in the study.

To conclude, for the first time, the present study developed a novel image processing algorithm which allows accurate and objective division of thyroid nodules into peripheral and central regions in ultrasound images. The algorithm also allows quantification of VI of the peripheral and central regions of thyroid nodules. The optimum offset level of dividing central and peripheral vascularity of thyroid nodules is 22%. Malignant thyroid nodules tend to be more vascular than benign nodules. The optimum cut-off of VI for overall, peripheral, and central vascularity in differentiating benign and malignant thyroid nodules were 20.2%, 19% and 9.1% respectively. The combination of VI assessment and GSU can improve the diagnostic accuracy for thyroid malignancy.

## Materials and Methods

This prospective study was approved by the Institutional Review Board of Prince of Wales Hospital. All study procedures and ultrasound examinations were performed in accordance with relevant guidelines and regulations. Informed written consent for the study was obtained from all patients. A total of 111 consecutive patients with one or more thyroid nodules (20 males, 91 females, mean age: 52.5 ± 13.6 years) were recruited from the Department of Surgery, Prince of Wales Hospital. The inclusion criteria for patient selection included presence of thyroid nodule(s) and availability of cytology/histology results of the nodule(s). Patients with associated Grave’s disease and Hashimoto diseases were excluded from the study. Patients with unconfirmed cytological or histological results of the nodules were also excluded.

### Ultrasound evaluation

All patients recruited in the study were arranged to have a GSU and color Doppler ultrasound examination of thyroid glands. Ultrasound examinations were performed by the same operator using the same ultrasound unit in conjunction with a 4–15 MHz linear transducer (SuperLinear™ SL15-4, Aixplorer, Supersonic Imagine, Aix-en-Provence, France). Standardized ultrasound scanning protocol was used for all patients. In the ultrasound examination, patient was asked to lie supine on the examination table with the shoulders supported by pillow. The patient’s neck was slightly hyperextended and the head was turned away from the side under examination. The transducer was gently placed over the neck for scanning without exerting compression on the patient’s neck. In each patient, the isthmus, right and left lobes of thyroid gland were assessed with transverse and longitudinal scans for detection of nodules. When a nodule was found, the grey scale sonographic features and vascularity of the nodule were assessed with GSU and color Doppler ultrasound respectively. In GSU, the thyroid nodule was assessed for the presence or absence of microcalcification, the margin regularity and the echogenicity. The margin of nodules was classified as regular or irregular. The echogenicity of nodules was categorized into hypoechoic, isoechoic and hyperechoic when compared to adjacent thyroid parenchyma. The tall to width ratio was also assessed by measuring the antero-posterior (i.e. tall) and medio-lateral (i.e. width) dimensions of the nodule in the transverse scan that showed the maximum cross-sectional area of the nodule. The tall to width ratio of nodules was categorized into ≤1 or >1.

In color Doppler ultrasound, the Doppler settings were standardized to high sensitivity with medium wall filter. The pulse repetition frequency (PRF) was set at 1000 Hz. The color gain was adjusted and standardized by an initial increase in the gain so that color noise appeared and was gradually decreased until the noise disappeared^21^. For each thyroid nodule, multiple transverse and longitudinal color Doppler ultrasound images were acquired.

All color Doppler sonograms of the thyroid nodule were analyzed quantitatively using a customized software algorithm. An independent observer performed the vascularity quantification analysis of thyroid nodules. The observer was unaware of the pathology results and final clinical diagnosis of the nodules. Sonograms were transferred from the ultrasound unit to a computer workstation installed with the MATLAB (version 7.3.0.267 R2006b; The Math Works, Natick, MA, USA). To evaluate the thyroid nodule vascularity, the nodule, i.e. region of interest (ROI), was manually outlined on the ultrasound image using Microsoft Paint (version 5.1: Microsoft Corporation, Redmond, W.A., USA). The image was then saved in tagged image file (TIF) format for further processing with MATLAB. Using our home-developed software algorithm in MATLAB, the number of color pixels and the total number of pixels within the ROI were counted. The vascularity index (VI) of the thyroid nodule was expressed as the percentage of the number of color pixels to the total number of pixels within the ROI. This calculation method was the same as described in our previous studies^21,25,26^. For each thyroid nodule, the color Doppler ultrasound image with the highest VI of the nodule was selected and used to evaluate the regional vascularity of the nodule.

**Figure 2:**
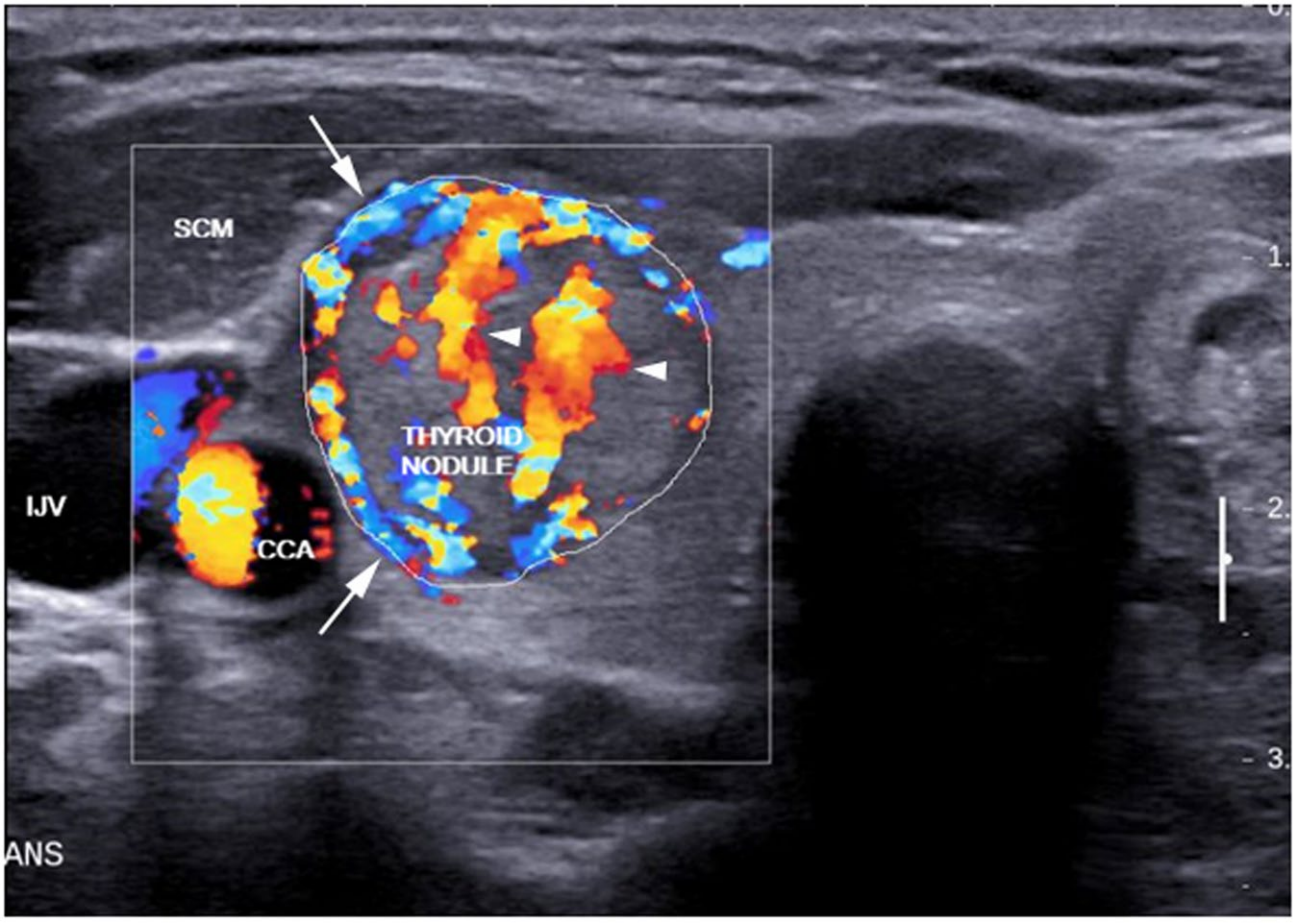
Transverse color Doppler sonogram of a nodule in the right thyroid lobe which was proven to be follicular thyroid carcinoma (outlined). The nodule showed both peripheral (arrows) and central (arrowheads) vascularity. SCM, sternocleidomastoid muscle; CCA, common carotid artery; IJV, internal jugular vein.

**Figure 3:**
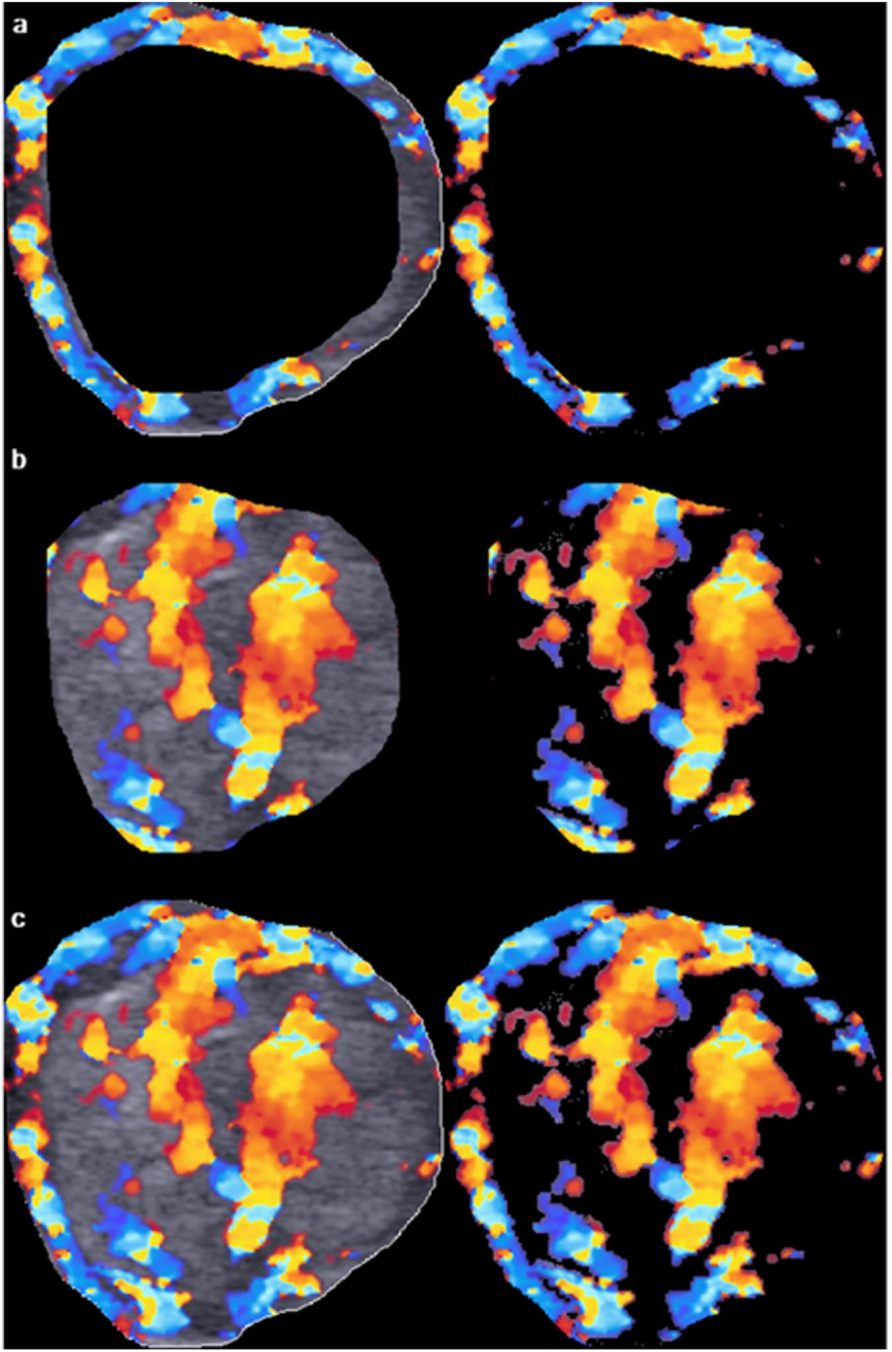
Image analysis of peripheral, central and overall vascular index (VI) of the thyroid nodule as shown in Fig. 2. The primary region of interest (ROI), i.e. the thyroid nodule, was extracted from the outlined area. Using an offset level of 10%, the peripheral (**a**, left) and central (**b**, left) regions of the nodule were segmented and the total number of pixel within the segmented area was counted by the computer algorithm. The color pixels coded by the color Doppler ultrasound were extracted by eliminating the grey scale pixels, and the color pixels were counted by the algorithm (**a**, right and **b**, right). The VI of peripheral and central regions of the nodule was the percentage of the number of color pixels to the total number of pixels within the segmented area. The overall VI of the nodule was evaluated by counting the total number of pixel within the ROI (**c**, left) and the number of color pixel in the image with the grey scale pixels eliminated (**c**, right).

To determine the VI of different regions of the nodule (i.e. peripheral and central), the primary ROI (i.e. the entire thyroid nodule, Fig. 2) was sub-divided into peripheral (Fig. 3a) and central (Fig. 3b) regions using the technique named “offsetting”. This sub-division of the nodule aimed to investigate the association of vascular distribution with the malignant status of the nodule. Figure 3c shows the evaluation of the overall vascularity of the entire nodule.

**Figure 4:**
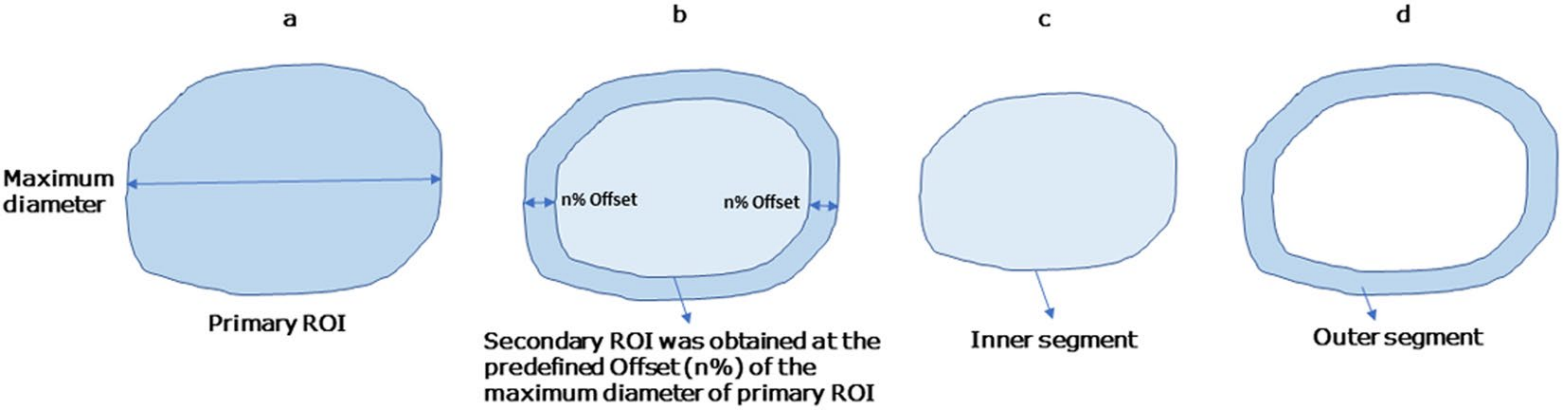
Schematic diagrams show the segmentation of peripheral and central regions of thyroid nodule. After the primary ROI was segmented by the algorithm, the maximum diameter of the ROI was identified (**a**). With the predefined offset level (n%) given to the algorithm, the secondary ROI was obtained (**b**). The secondary ROI represents the inner segment (i.e. central region) of the nodule (**c**). The outer segment (i.e. peripheral region) of the nodule was obtained by subtracting the primary ROI by the secondary ROI (**d**).

“Offsetting” is used to increase or decrease the area of arbitrary shapes without distorting the shape and keep the contour of the ROI. The inner segment (i.e. secondary ROI = central region of the primary ROI) is an inward offset (an operation also known as “deflating” or “buffering” of the primary ROI). The outer segment is obtained by subtracting the primary ROI by the secondary ROI. The magnitude of offset was varied as a percentage of the maximum diameter of the primary ROI (Fig. 4).

Rather than treating the ROI as a polygon for mathematical offsetting, our algorithm uses a raster-image based approach that iteratively erodes the primary ROI with a small kernel. This approach produces results with the benefit of not having to implement polygon clipping in MATLAB so that the contour of the secondary ROI (i.e. the inner segment of the nodule after offsetting) is smoother and similar to the contour of the primary ROI (i.e. the thyroid nodule).

The optimum offset was determined by exploring the difference in VI between benign and malignant nodules in both inner (i.e. central) and outer (i.e. peripheral) segments. In the study, we tried a range of offsets with an interval of 5% (i.e. 5%,10%,15%,20% and 25%). We did not exceed offsetting from 25% because previous literature suggested that central region of a thyroid nodule is considered to be the inner 90% of the diameter of the nodule^2^. At each offset level, the VI of the peripheral and central regions of the benign and malignant nodules were measured. The offset level that resulted in a significant difference (*P* < 0.05) in VI between benign and malignant nodules in both central and peripheral regions was considered to be the optimum offset level.

The significance of the difference of grey-scale ultrasound features between benign and malignant thyroid nodules was evaluated using Chi-square test. Student independent t-test was used to calculate the significance of the difference of VI between benign and malignant thyroid nodules. Receiver operating characteristic (ROC) curves were used to determine the optimum cut-off of VI in differentiating benign and malignant nodules, and the associated diagnostic performance at the optimum cut-off. All statistical analyses were performed using the Statistical Package for the Social Sciences (SPSS) software (Version 24, IBM Corporation, Armonk, NY, USA). Two-tailed *P* < 0.05 was considered significant.

### Data availability statement

The datasets generated during and/or analysed during the current study are available from the corresponding author on reasonable request.
